# Afabicin, a First-in-Class Antistaphylococcal Antibiotic, in the Treatment of Acute Bacterial Skin and Skin Structure Infections: Clinical Noninferiority to Vancomycin/Linezolid

**DOI:** 10.1128/AAC.00250-20

**Published:** 2020-09-21

**Authors:** Frederick Wittke, Catherine Vincent, James Chen, Barry Heller, Heidi Kabler, J. Scott Overcash, François Leylavergne, Guennaëlle Dieppois

**Affiliations:** a Debiopharm International SA, Lausanne, Switzerland; b Southbay Pharma Research, Buena Park, California, USA; c Long Beach Clinical Trials, Long Beach, California, USA; d Sunrise Hospital and Medical Center, Las Vegas, Nevada, USA; e eStudySite, San Diego, California, USA

**Keywords:** ABSSSI, FabI inhibitor, staphylococcus, afabicin, efficacy, phase II, safety

## Abstract

Afabicin (formerly Debio 1450, AFN-1720) is a prodrug of afabicin desphosphono, an enoyl-acyl carrier protein reductase (FabI) inhibitor, and is a first-in-class antibiotic with a novel mode of action to specifically target fatty acid synthesis in *Staphylococcus* spp. The efficacy, safety, and tolerability of afabicin were compared with those of vancomycin/linezolid in the treatment of acute bacterial skin and skin structure infections (ABSSSI) due to staphylococci in this multicenter, parallel-group, double-blind, and double-dummy phase 2 study. Randomized patients (1:1:1) received either low-dose (LD) afabicin (intravenous [i.v.] 80 mg, followed by oral 120 mg, twice a day [BID]), high-dose (HD) afabicin (i.v. 160 mg, followed by oral 240 mg, BID), or vancomycin/linezolid (i.v. vancomycin 1 g or 15 mg/kg, followed by oral linezolid 600 mg, BID). The most frequent baseline pathogen was Staphylococcus aureus (97.5% of microbiological intent-to-treat [mITT] population), and 50.4% of patients had methicillin-resistant S. aureus. Clinical response rates at 48 to 72 h postrandomization in the mITT population were comparable among treatment groups (94.6%, 90.1%, and 91.1%, respectively). Both LD and HD afabicin were noninferior to vancomycin/linezolid (differences, −3.5% [95% confidence interval {CI}, −10.8%, 3.9%] and 1.0% [95% CI, −7.3%, 9.2%], respectively). Most common treatment-emergent adverse events were mild and were headache (9.1% and 16.8%) and nausea (6.4% and 8.4%) with LD and HD afabicin, respectively. Afabicin was efficacious and well tolerated in the treatment of ABSSSI due to staphylococci, and these data support further development of afabicin for the treatment of ABSSSI and potentially other types of staphylococcal infections. (This study has been registered at ClinicalTrials.gov under identifier NCT02426918.)

## Abstract (precis)

Afabicin (formerly Debio 1450, AFN-1720) is a prodrug of afabicin desphosphono, an enoyl-acyl carrier protein reductase (FabI) inhibitor, and is a first-in-class antibiotic with a novel mode of action to specifically target fatty acid synthesis in *Staphylococcus* spp. The efficacy, safety, and tolerability of afabicin were compared with those of vancomycin/linezolid in the treatment of acute bacterial skin and skin structure infections (ABSSSI) due to staphylococci in this multicenter, parallel-group, double-blind, and double-dummy phase 2 study.

## INTRODUCTION

Staphylococcus aureus is the predominant bacterial pathogen responsible for a wide range of infections, including acute bacterial skin and skin structure infections (ABSSSI), infective endocarditis, osteoarticular infections, and prosthetic device infections (1). Antibiotics belonging to several chemical classes, including β-lactams, lipopeptides, glycopeptides, and oxazolidinones, are available for the treatment of staphylococcal infections (1–3); however, many have reduced efficacy due to increasing or, for newer antibiotics, emerging antibiotic resistance. There are concerns with a number of the approved agents for the treatment of ABSSSI, including safety, lack of oral formulation, and emerging resistance, which can limit their use (1, 4–7), underscoring the need for extending the available treatment options for this indication. In addition, the use of broad-spectrum antibiotics is likely to disturb the normal intestinal microbiota (8), which can result in antibiotic-induced complications such as colitis and candidiasis, and contribute to the spread of antibiotic resistance genes and selection of multidrug-resistant pathogens (9–12). However, species-targeted antimicrobial agents may reduce off-target selection pressures on the human microbiota (13). It is therefore essential to develop new antibiotics with activity against isolates resistant to current antibiotics and which have minimal impact on the microbiota.

Afabicin (formerly Debio 1450, AFN-1720) is a first-in-class antibiotic with a novel mode of action to specifically target fatty acid synthesis in *Staphylococcus* spp., including antibiotic-resistant strains, and minimize impact on the intestinal microbiota. Afabicin is a prodrug of afabicin desphosphono (formerly Debio 1452, AFN-1252), an enoyl-acyl carrier protein reductase FabI inhibitor. The development of afabicin, by enhancing the solubility of afabicin desphosphono, has provided both intravenous (i.v.) and oral formulations of the antibiotic. Following i.v. or oral dosing, afabicin is rapidly converted to its active moiety, as demonstrated in phase 1 studies (14).

The efficacy and safety of afabicin desphosphono were investigated in an earlier open-label, noncomparative, phase 2 study in patients with ABSSSI, including patients with significant comorbidities (15). Following oral administration of afabicin desphosphono at 200 mg twice daily (BID), 82.9% of patients in the microbiologically evaluable population achieved a ≥20% decrease in the area of erythema on day 3.

The objective of the current study was to investigate the efficacy and safety of i.v. and oral afabicin prodrug at two dose levels, in comparison with i.v. vancomycin/oral linezolid, in the treatment of ABSSSI.

## RESULTS

### Patients.

**FIG 1:**
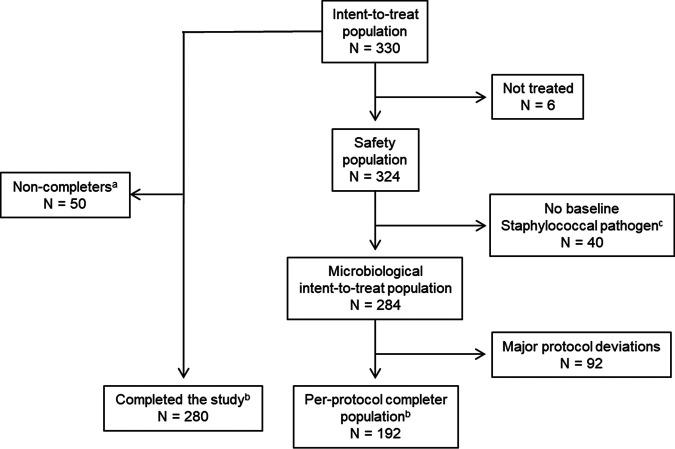
Patient disposition. ^a^, For those indicated as “Non-completers,” reasons for noncompletion included adverse events (4 patients, including 3 patients who had a serious adverse event and 1 patient who died [see “Safety”]), physician decision (3 patients, including 1 patient who was not treated, 1 patient in the LD afabicin group who was withdrawn on day 2, and 1 patient in the vancomycin/linezolid group who was withdrawn on day 28), withdrawal by patient (14 patients), and other reasons (29 patients). ^b^, For those indicated as having “Completed the study” and the “Per-protocol completer population,” these patients completed up to STFU. ^c^, For those for which there was “No baseline staphylococcal pathogen,” the patients met the criteria for inclusion, but pathogenic staphylococcal species were not identified from baseline lesion or blood samples by the central laboratory.

A total of 330 patients were randomized at 15 study centers in the United States (Fig. 1). The intent-to-treat (ITT) population comprised 330 patients, of which 280 (84.8%) completed the study up to short-term follow-up (STFU). The microbiological ITT (mITT) population comprised 284 patients (*n* = 92, *n* = 91, and *n* = 101 in the low-dose [LD] afabicin, high-dose [HD] afabicin, and vancomycin/linezolid groups, respectively), and the per-protocol (PP) population included 192 patients (*n* = 67, *n* = 57, and *n* = 68 in the LD afabicin, HD afabicin, and vancomycin/linezolid groups, respectively).

In the mITT population, the demographic and baseline characteristics were comparable among the three treatment groups, although in the vancomycin/linezolid group, the percentage of male patients and the percentage of patients with wound infections were slightly higher and the mean areas of the primary lesions were slightly larger (Table 1).

**TABLE 1:** Demography and baseline disease characteristics (mITT population)^*a*^

Variable	Value for indicated treatment group		
	LD afabicin (80/120 mg BID) (*n* = 92)	HD afabicin (160/240 mg BID) (*n* = 91)	Vancomycin/linezolid (BID) (*n* = 101)
Age (yr)			
Mean (SD)	43.8 (11.9)	42.3 (11.7)	44.9 (10.6)
Median (min, max)	44.5 (18, 69)	42.0 (21, 65)	46.0 (23, 64)
Sex, *n* (%)			
Male	61 (66.3)	60 (65.9)	73 (72.3)
Body wt (kg)			
Mean (SD)	77.3 (15.5)	77.9 (18.9)	83.3 (20.4)
Median (min, max)	75.35 (52.7, 124.5)	74.9 (41.3, 157.0)	78.0 (53.9, 154.0)
BMI (kg/m^2^)			
Mean (SD)	26.8 (4.9)	26.5 (6.3)	28.5 (7.7)
Median (min, max)	26.2 (16.6, 43.1)	25.3 (11.2, 61.3)	26.4 (19.1, 64.5)
≥30 kg/m^2^, *n* (%)	22 (23.9)	19 (20.9)	32 (31.7)
Infection type, *n* (%)			
Cellulitis	18 (19.6)	15 (16.5)	18 (17.8)
Noncellulitis	74 (80.4)	76 (83.5)	83 (82.2)
Lesion type, *n* (%)			
Wound	52 (56.5)	51 (56.0)	64 (63.4)
Major abscess	29 (31.5)	29 (31.9)	24 (23.8)
Cellulitis	11 (12.0)	11 (12.1)	13 (12.9)
Burn	0	0	0
Lesion area, cm²			
Mean (SD)	338.9 (240.8)	332.1 (269.4)	375.9 (252.5)
Median (min, max)	292.7 (76.5, 1394.0)	270.6 (78.0, 1932.0)	321.2 (76.5, 1200.5)
Diabetes mellitus, *n* (%)			
Presence	6 (6.5)	6 (6.6)	6 (5.9)
History of drug abuse, *n* (%)			
Yes	77 (83.7)	76 (83.5)	83 (82.2)

a BID, twice daily; BMI, body mass index; HD, high dose; LD, low dose; mITT, microbiological intent-to-treat; *n*, total number of patients; SD, standard deviation; min, minimum; max, maximum.

By definition, S. aureus or a pathogenic coagulase-negative staphylococcus (CoNS) was isolated at baseline from the primary lesion or blood of all patients in the mITT population. Overall, S. aureus was isolated from 277 (97.5%) patients; the percentages of methicillin-resistant S. aureus (MRSA) and methicillin-susceptible S. aureus (MSSA) isolates were similar and comparable among treatment groups (Table 2). Pathogenic CoNS, most commonly Staphylococcus epidermidis, were isolated from 11 patients (3.9%). Four patients were coinfected with S. aureus and a pathogenic CoNS. The most commonly isolated nonstaphylococcal Gram-positive pathogens at baseline were Streptococcus pyogenes (9 patients), Streptococcus constellatus (7 patients), and Streptococcus anginosus (6 patients) (Table 2), and coinfection with a staphylococcal and a streptococcal species was identified in 11.3% of patients. The most commonly isolated Gram-negative pathogens at baseline were Klebsiella pneumoniae (9 patients), Klebsiella oxytoca (6 patients), and Enterobacter cloacae (6 patients). Coinfection with a staphylococcal and a nonstaphylococcal species was identified in the primary lesions of 21.1% (60/284) of patients (Table 2).

**TABLE 2:** Microbiological profile: patients with most frequent baseline pathogens isolated from the primary lesion or blood at baseline (mITT population)^*a*^

Baseline pathogen	No. (%) of patients with pathogen(s)^*b*^ in indicated treatment group		
	LD afabicin (80/120 mg BID) (*n* = 92)	HD afabicin (160/240 mg BID) (*n* = 91)	Vancomycin/linezolid (BID) (*n* = 101)
Staphylococcus aureus^*c*^	90 (97.8)	88 (96.7)	99 (98.0)
MRSA	45 (48.9)	46 (50.5)	52 (51.5)
MSSA	46 (50.0)	42 (46.2)	47 (46.5)
Pathogenic CoNS^*c*^	6 (6.5)	3 (3.3)	2 (2.0)
Staphylococcus epidermidis	5 (5.4)	3 (3.3)	2 (2.0)
Staphylococcus haemolyticus	0	1 (1.1)	0
Staphylococcus lugdunensis	1 (1.1)	0	0
Nonpathogenic CoNS	4 (4.3)	0	0
Staphylococcus capitis	1 (1.1)	0	0
Staphylococcus hominis	3 (3.3)	0	0
*Streptococcus* spp.			
S. agalactiae	1 (1.1)	0	2 (2.0)
*S. anginosus*	1 (1.1)	3 (3.3)	2 (2.0)
*S. constellatus*	3 (3.3)	1 (1.1)	3 (3.0)
S. intermedius	0	3 (3.3)	1 (1.0)
S. pyogenes	3 (3.3)	0	6 (5.9)
Other *Streptococcus* spp.	2 (2.2)^*d*^	1 (1.1)^*e*^	2 (2.0)^*f*^
Enterococcus faecalis	1 (1.1)	0	2 (2.0)
*Klebsiella* spp.			
K. oxytoca	3 (3.3)	3 (3.3)	0
K. pneumoniae	4 (4.3)	1 (1.1)	4 (4.0)
Enterobacter cloacae	1 (1.1)	2 (2.2)	3 (3.0)
*Prevotella* spp.	1 (1.1)	0	3 (3.0)

a BID, twice daily; HD, high dose; LD, low dose; mITT, microbiological intent-to-treat; MRSA, methicillin-resistant Staphylococcus aureus; MSSA, methicillin-susceptible Staphylococcus aureus; *n*, total number of patients.

b Most frequent pathogens isolated from the primary lesion or blood at baseline. A patient may have more than one pathogen isolated.

c By definition, all patients in the mITT population were culture positive for S. aureus and/or a pathogenic CoNS.

d *S. gallolyticus*, 1 patient; *S. massiliensis* and S. mutans, 1 patient.

e *S. oralis*, 1 patient.

f *S. equi*, 1 patient; beta-hemolytic streptococci, 1 patient.

The afabicin desphosphono (active moiety of afabicin) MIC_50_ and MIC_90_ values for both MSSA and MRSA isolates collected at baseline were 0.008 mg/liter and ≤0.015 mg/liter, respectively. All S. aureus isolates were susceptible to vancomycin (MIC ≤ 1 mg/liter) and linezolid (MIC ≤ 2 mg/liter).

The overall mean durations of i.v. and oral treatment in the mITT population were 1.1 days (means ranged from 1.0 days for HD afabicin to 1.2 days for LD afabicin) and 6.6 days (mean, 6.6 days for all treatment groups), respectively. Concomitant antibiotics were used more frequently in the afabicin treatment groups (23.9% and 24.2% of patients) than in the vancomycin/linezolid treatment groups (16.8%). Amoxicillin was the most common concomitant antibiotic, administered to 13.0%, 12.1%, and 13.9% of patients in the LD afabicin, HD afabicin, and vancomycin/linezolid treatment groups, respectively. The use of short-acting antibiotics within 24 h prior to randomization was infrequent (5.3% of patients overall). Of the patients with polymicrobial infections with a staphylococcal and a nonstaphylococcal species, 23.5% (8/34) of patients in the afabicin groups and 30.8% (8/26) of patients in the vancomycin/linezolid groups received a concomitant antibiotic.

### Primary efficacy outcome.

The primary efficacy outcomes of early clinical response at 48 to 72 h postrandomization, as specified in the FDA guidelines (16), were comparable among treatment groups (94.6%, 90.1%, and 91.1% for LD afabicin, HD afabicin, and vancomycin/linezolid, respectively) in the mITT population (Table 3). Both LD afabicin and HD afabicin were found to be noninferior to vancomycin/linezolid (difference, −3.5% [95% confidence interval {CI}, −10.8 to 3.9%] for LD afabicin; difference, 1.0% [95% CI , −7.3 to 9.2%] for HD afabicin). There were 23 patients that had not responded to treatment at the primary endpoint (*n* = 5, *n* = 9, and *n* = 9 for LD afabicin, HD afabicin, and vancomycin/linezolid, respectively) (Table 3).

**TABLE 3:** Early clinical response (primary efficacy endpoint) and clinical outcomes (secondary endpoints)^*a*^

Variable	Value for indicated treatment group		
	LD afabicin (80/120 mg BID)	HD afabicin (160/240 mg BID)	Vancomycin/linezolid (BID)
mITT population	*n* = 92	*n* = 91	*n* = 101
Early clinical response			
Responder, *n* (%)	87 (94.6)	82 (90.1)	92 (91.1)
Nonresponders, *n* (%)	5 (5.4)^*b*^	9 (9.9)^*c*^	9 (8.9)^*d*^
Treatment difference vs VAN/LZD, %	−3.5	1.0	
95% CI	(−10.8, 3.9)	(−7.3, 9.2)	
Noninferiority	Yes	Yes	
Clinical outcome 48 to 72 h postrandomization			
Success, *n* (%)	77 (83.7)	74 (81.3)	87 (86.1)
Failure, *n* (%)	15 (16.3)	17 (18.7)	14 (13.9)
of which indeterminates, *n*	3	8	3
95% CI for success	74.5, 90.6	71.8, 88.7	77.8, 92.2
95% CI for failure	9.4, 25.5	11.3, 28.2	7.8, 22.2
Clinical outcome at EOT			
Success, *n* (%)	85 (92.4)	80 (87.9)	93 (92.1)
Failure, *n* (%)	7 (7.6)	11 (12.1)	8 (7.9)
of which indeterminates, *n*	5	7	6
95% CI for success	84.95, 96.9	79.4, 93.8	85.0, 96.5
95% CI for failure	3.1, 15.05	6.2, 20.6	3.5, 15.0
Clinical outcome at STFU			
Success, *n* (%)	78 (84.8)	76 (83.5)	93 (92.1)
Failure, *n* (%)	14 (15.2)	15 (16.5)	8 (7.9)
of which indeterminates, *n*	10	10	7
95% CI for success	75.8, 91.4	74.3, 90.5	85.0, 96.5
95% CI for failure	8.6, 24.2	9.5, 25.7	3.5, 15.0
			
PP population	*n* = 67	*n* = 57	*n* = 68
Clinical outcome 48 to 72 h postrandomization			
Success, *n* (%)	58 (86.6)	50 (87.7)	62 (91.2)
95% CI for success	76.0, 93.7	76.3, 94.9	81.8, 96.7
Failure, *n* (%)	9 (13.4)	7 (12.3)	6 (8.8)
95% CI for failure	6.3, 24.0	5.1, 23.7	3.3, 18.2
Clinical outcome at EOT			
Success, *n* (%)	65 (97.0)	55 (96.5)	67 (98.5)
95% CI for success	89.6, 99.6	87.9, 99.6	92.1, 100.0
Failure, *n* (%)	2 (3.0)	2 (3.5)	1 (1.5)
95% CI for failure	0.4, 10.4	0.4, 12.1	0.04, 7.9
Clinical outcome at STFU			
Success, *n* (%)	65 (97.0)	56 (98.2)	68 (100.0)
95% CI for success	89.6, 99.6	90.6, 100.0	94.7, 100.0
Failure, *n* (%)	2 (3.0)	1 (1.8)	0
95% CI for failure	0.4, 10.4	0.04, 9.4	0.00, 5.3

a BID, twice daily; CI, confidence interval; EOT, end of treatment; HD, high dose; LD, low dose; mITT, microbiological intent-to-treat; *n*, total number of patients; PP, per protocol; STFU, short-term follow-up; VAN/LZD, vancomycin/linezolid.

b Baseline pathogens: MSSA only, 1 patient; MRSA only, 4 patients.

c Baseline pathogens: MSSA only, 5 patients; MRSA only, 4 patients.

d Baseline pathogens: MSSA only, 1 patient; MRSA only, 4 patients; MSSA, Streptococcus constellatus, and Prevotella nigrescens, 1 patient; MSSA, Streptococcus agalactiae, Enterococcus faecalis, Citrobacter koseri, Enterobacter cloacae, and Pseudomonas stutzeri, 1 patient; Staphylococcus epidermidis, Clostridium perfringens, 1 patient; MRSA and Streptococcus pyogenes, 1 patient.

All patients with polymicrobial infections involving a nonstaphylococcal pathogen in the afabicin groups (*n* = 19 in the LD group, *n* = 15 in the HD group) were responders for the primary endpoint at 48 to 72 h postrandomization, compared with 22/26 in the vancomycin/linezolid group (Table 4). In the LD and HD afabicin groups, the baseline pathogen of nonresponders was S. aureus only. In the vancomycin/linezolid group, baseline pathogens were either S. aureus only or polymicrobial (see footnotes in Table 3). Among the patients with polymicrobial infections in the afabicin groups, 16 were infected with Gram-positive species only (*n* = 8 in the LD group, *n* = 8 in the HD group) and 18 were infected with Gram-positive and Gram-negative species (*n* = 11 in the LD group, *n* = 8 in the HD group). In the vancomycin/linezolid group, 10 patients were infected with Gram-positive species only and 12 patients were coinfected with Gram-positive and Gram-negative species (Table 5).

**TABLE 4:** Early clinical response in the monomicrobial and polymicrobial mITT populations: monomicrobial population versus polymicrobial population

Population and treatment	No. (%) of patients		
	Nonresponder	Responder	Total
Monomicrobial population (mITT subjects with *Staphylococcus* species only)
Vancomycin/linezolid, BID	5 (6.7)	70 (93.3)	75 (33.5)
Afabicin, 80 mg/120 mg BID	5 (6.9)	68 (93.1)	73 (32.6)
Afabicin, 160 mg/240 mg BID	9 (11.8)	67 (88.2)	76 (33.9)
Total monomicrobial population	19 (8.5)	205 (91.5)	224 (100)
			
Polymicrobial population (mITT subjects with *Staphylococcus* species and other bacterial species)			
Vancomycin/linezolid, BID	4 (15.4)	22 (84.6)	26 (43.3)
Afabicin, 80 mg/120 mg BID	0 (0)	19 (100)	19 (30)
Afabicin, 160 mg/240 mg BID	0 (0)	15 (100)	15 (26.7)
Total polymicrobial population	4 (6.7)	56 (93.3)	60 (100)
			
Total mITT population	23 (8.1)	261 (91.9)	284 (100)

**TABLE 5:** Early clinical response in the monomicrobial and polymicrobial mITT populations: polymicrobial population infected with Gram-positive species versus polymicrobial population coinfected with Gram-negative species

Population and treatment^*a*^	No. (%) of patients		
	Nonresponder	Responder	Total
Polymicrobial population Gram+ only (mITT subjects with *Staphylococcus* species and other Gram+ species only)
Vancomycin/linezolid, BID	2 (16.7)	10 (83.3)	12 (42.8)
Afabicin, 80 mg/120 mg BID	0 (0)	8 (100)	8 (28.6)
Afabicin, 160 mg/240 mg BID	0 (0)	8 (100)	8 (28.6)
Total with only *Staphylococcus*	2 (7.1)	26 (92.9)	28 (100)
			
Polymicrobial population Gram− (mITT subjects with *Staphylococcus* species and Gram− species)			
Vancomycin/linezolid, BID	2 (14.3)	12 (85.7)	14 (43.7)
Afabicin, 80 mg/120 mg BID	0 (0)	11 (100)	11 (34.4)
Afabicin, 160 mg/240 mg BID	0 (0)	7 (100)	7 (21.9)
Total with *Staphylococcus* and Gram−	2 (6.3)	30 (93.7)	32 (100)
			
Total polymicrobial population	4 (6.7)	56 (93.3)	60 (100)

a Gram+, Gram positive; Gram−, Gram negative.

### Secondary outcomes.

The secondary efficacy outcomes of clinical outcomes at 48 to 72 h postrandomization, end of treatment (EOT), and STFU are presented in Table 3. At 48 to 72 h, clinical success rates were comparable between treatment groups. The clinical success rates at EOT were similar in the LD afabicin and vancomycin/linezolid groups; however, at STFU, the rate was higher in the vancomycin/linezolid group (92.1%) than in the LD afabicin and HD afabicin groups (84.8% and 83.5%, respectively). The clinical success rates at EOT were marginally higher than at 48 to 72 h postrandomization. At STFU, clinical success rates in the afabicin groups were comparable to the rates at 48 to 72 h postrandomization; however, in the vancomycin/linezolid group, the rate at STFU was higher than that at 48 to 72 h postrandomization (86.1%) and the same as that at EOT (92.1%). Among the patients who met the primary efficacy endpoint of early clinical response, 88.5%, 90.2%, and 96.7% in the LD afabicin, HD afabicin, and vancomycin/linezolid groups, respectively, were categorized as clinical successes at STFU.

Clinical failure rates at 48 to 72 h postrandomization were slightly lower in the vancomycin/linezolid group (13.9%) than in the LD afabicin (16.3%) and HD afabicin (18.7%) groups (Table 3). For each of the treatment groups, the most frequent reason for clinical failure was the requirement of further antibiotic treatment of the original site of infection, due to new signs, symptoms, or complications attributable to the ABSSSI (12.0%, 8.8%, and 7.9% in the LD afabicin, HD afabicin, and vancomycin/linezolid groups, respectively).

Clinical success rates in the PP population were marginally higher than in the mITT population, and the largest differences were seen at STFU (Table 3).

Few postbaseline samples were taken from skin lesions: *n* = 54 at 48 to 72 h postrandomization; *n* = 5 at EOT; and *n* = 2 at STFU. Therefore, microbiological eradication rates presented in Table 6 were based largely on the investigator’s assessment of clinical outcome. At 48 to 72 h postrandomization, of the 54 patients who had a lesion sample taken, 5/17 in the LD afabicin group, 3/15 in the HD afabicin group, and 6/22 in the vancomycin/linezolid group had a microbiological outcome of documented eradication. Of these, two patients had a superinfection at 48 to 72 h postrandomization: one patient in the LD afabicin group (MSSA at baseline, MRSA at 48 to 72 h postrandomization) and one patient in the vancomycin/linezolid group (MRSA and S. constellatus at baseline, S. epidermidis at 48 to 72 h postrandomization). No decrease in afabicin desphosphono activity has been observed in the collected isolates compared with that at baseline.

**TABLE 6:** Microbiological eradication rate by baseline pathogen (mITT population) 48 to 72 h postrandomization^*a*^

Baseline pathogen, by patient	No. of patients with microbiological eradication/total no. (%)^*b*^		
	LD afabicin (80/120 mg BID) (*n* = 92)	HD afabicin (160/240 mg BID) (*n* = 91)	Vancomycin/linezolid (BID) (*n* = 101)
Staphylococcus aureus	65/90 (72.2)	60/88 (68.2)	75/99 (75.8)
MSSA	35/46 (76.1)	28/42 (66.7)	36/47 (76.6)
MRSA	31/45 (68.9)	32/46 (69.6)	39/52 (75.0)
			
Pathogenic CoNS	6/6 (100.0)	3/3 (100.0)	1/2 (50.0)
Staphylococcus epidermidis	5/5 (100.0)	3/3 (100.0)	1/2 (50.0)
Staphylococcus haemolyticus	0/0	1/1 (100.0)	0/0
Staphylococcus lugdunensis	1/1 (100.0)	0/0	0/0

a BID, twice daily; CoNS, coagulase-negative staphylococci; HD, high dose; LD, low dose; mITT, microbiological intent-to-treat; MSSA, methicillin-susceptible Staphylococcus aureus; MRSA, methicillin-resistant Staphylococcus aureus; *n*, total number of patients.

b Eradication rates are based on presumed and documented eradication.

To evaluate the clinical response, the area of the primary lesion was measured at the screening visit to provide a baseline value, at the primary endpoint of 48 to 72 h after the randomization, at EOT, and at STFU. A summary of lesion area and change from baseline in the mITT population is presented in Table 7. Overall, the mean (SD) lesion area at baseline by ruler and digital photography was 349.889 (254.2175) cm^2^ and 241.383 (168.9697) cm^2^, respectively. The changes from baseline were comparable across the three treatment groups. Overall, the maximum change from baseline by ruler was observed at STFU (approximately 98%). The mean (SD) change from baseline by ruler at STFU was −99.221 (3.0001), −98.433 (7.2591), and −98.635 (4.7646) cm^2^, respectively, for the low-dose afabicin group, high-dose afabicin group, and control group. The percentages of change from baseline in lesion area over time by ruler and digital photography were comparable across the three treatment groups.

**TABLE 7:** Summary of lesion area and change from baseline using ruler (mITT population)^*a*^

Parameter	Statistic	Value for treatment group			Overall value (*n* = 284)
		Afabicin, 80 mg/120 mg BID (*n* = 92)	Afabicin, 160 mg/240 mg BID (*n* = 91)	Vancomycin/linezolid, BID (*n* = 101)	
	*n*	92	91	101	284
Baseline lesion area, cm^2^	Mean (SD)	338.877 (240.7652)	332.141 (269.4060)	375.911 (252.4650)	349.889 (254.2175)
	Median	292.720	270.600	321.180	280.940
	Min, max	76.50, 1,394.00	78.00, 1,932.00	76.50, 1,200.50	76.50, 1,932.00
					
	*n*	89	84	98	271
Lesion area at 48 to 72 h after randomization, cm^2^	Mean (SD)	123.087 (131.5498)	109.209 (157.3943)	144.461 (200.8444)	126.515 (167.2171)
	Median	91.800	82.260	88.185	86.130
	Min, max	4.00, 891.54	0.00, 1,328.00	0.00, 1,401.60	0.00, 1,401.60
					
	*n*	89	84	98	271
Change from baseline, %	Mean (SD)	−64.057 (22.8976)	−66.207(22.1466)	−64.265 (27.1928)	−64.799 (24.2531)
	Median	−68.627	−71.302	−70.175	−69.629
	Min, max	−99.11, −12.16	−100.00, 0.00	−100.00, 51.79	−100.00, 51.79
					
	*n*	86	85	95	266
Lesion area at EOT– early termination, cm^2^	Mean (SD)	20.886 (41.1057)	23.183 (40.8145)	22.555 (42.1446)	22.216 (41.2427)
	Median	2.205	8.750	5.000	5.000
	Min, max	0.00, 263.16	0.00, 218.12	0.00, 216.00	0.00, 263.16
					
	*n*	86	85	95	266
Change from baseline, %	Mean (SD)	−93.715 (11.1415)	−97.497 (12.0164)	−98.528 (13.5691)	−98.298 (12.3045)
	Median	−98.648	−97.497	−98.528	−98.298
	Min, max	−100.00, −32.72	−100.00, −48.00	−100.00, −11.38	−100.00, −11.38
					
	*n*	81	81	94	256
STFU lesion area, cm^2^	Mean (SD)	2.645 (11.0402)	2.769 (10.3083)	3.708 (12.3967)	3.074 (11.3075)
	Median	0.000	0.000	0.000	0.000
	Min, max	0.00, 89.25	0.00, 72.00	0.00, 71.50	0.00, 89.25
					
	*n*	81	81	94	256
Change from baseline, %	Mean (SD)	−99.221 (3.0001)	−98.433 (7.2591)	−98.635 (4.7646)	−98.757 (5.2671)
	Median	−100.00	−100.00	−100.00	−100.00
	Min, max	−100.00, −75.21	−100, −40.00	−100, −69.57	−100.00, −40.00

a BID, twice daily; mITT, microbiological intent-to-treat; *n*, total number of patients; SD, standard deviation; min, minimum; max, maximum.

As part of secondary endpoints, the clinical success was assessed for different lesion types, i.e., wound, major abscess, and cellulitis. However, the comparison did not show statistically significant differences in success rates (Table 8).

**TABLE 8:** Secondary efficacy analysis: summary of investigator's assessment of clinical outcome by stratification factors (mITT population)^*a*^

Investigator’s assessment of clinical outcome by lesion type	Statistic	Value for treatment group			Overall value (*n* = 284)
		Afabicin, 80 mg/120 mg BID (*n* = 92)	Afabicin, 160 mg/240 mg BID (*n* = 91)	Vancomycin/linezolid, BID (*n* = 101)	
Wound	*n*	52	51	64	167
48 to 72 h after randomization					
Success	*n* (%)	45 (86.5)	38 (74.5)	52 (81.3)	135 (80.8)
Failures	*n* (%)	7 (13.5)	13 (25.5)	12 (18.8)	32 (19.2)
thereof indeterminates		1	7	2	10
95% CI for the success rate		74.21, 94.41	60.37, 85.67	69.54, 89.92	74.04, 86.51
95% CI for the failure rate		5.59, 25.79	14.33, 39.63	10.08, 30.46	13.49, 25.96
EOT					
Success	*n* (%)	48 (92.3)	43 (84.3)	59 (92.2)	150 (89.8)
Failures	*n* (%)	4 (7.7)	8 (7.8)	5 (7.8)	17 (10.2)
thereof indeterminates		3	4	3	10
95% CI for success rate		81.46, 97.86	71.41, 92.98	82.70, 97.41	84.20, 93.96
95% CI for failure rate		2.14, 18.54	7.02, 28.59	2.59, 17.30	6.04, 15.80
STFU					
Success	*n* (%)	44 (84.6)	42 (82.4)	61 (95.3)	147 (88.0)
Failures	*n* (%)	8 (15.4)	9 (17.6)	3 (4.7)	20 (12.0)
thereof indeterminates		6	5	2	13
95% CI for success rate		71.92, 93.12	69.13, 91.60	86.91, 99.02	82.11, 92.53
95% CI for failure rate		6.88, 28.08	8.40, 30.87	0.98, 13.09	7.47, 17.89
Abscess	*n*	29	29	24	82
48 to 72 h after randomization					
Success	*n* (%)	23 (79.3)	26 (89.7)	23 (95.8)	72 (87.8)
Failures	*n* (%)	6 (20.7)	3 (10.3)	1 (4.2)	10 (12.2)
thereof indeterminates	*n*	1	0	0	1
95% CI for success rate		60.28, 92.01	72.65, 97.81	78.88, 99.89	78.71, 93.99
95% CI for failure rate		7.99, 39.72	2.19, 27.35	0.11, 21.12	6.01, 21.29
EOT					
Success	*n* (%)	27 (93.1)	21 (93.1)	22 (91.7)	76 (92.7)
Failures	*n* (%)	2 (6.9)	2 (6.9)	2 (8.3)	6 (7.3)
thereof indeterminates	*n*	1	2	2	5
95% CI for success rate		77.23, 99.15	77.23, 99.15	73.00, 98.97	84.75, 97.27
95% CI for failure rate		0.85, 22.77	0.85, 22.77	1.03, 27.00	2.73, 15.25
STFU					
Success	*n* (%)	25 (86.2)	26 (89.7)	21 (87.5)	72 (87.8)
Failures	*n* (%)	4 (13.8)	3 (10.3)	3 (12.5)	10 (12.2.)
thereof indeterminates	*n*	3	3	3	9
95% CI for success rate		68.34, 96.11	72.65, 97.81	67.64, 97.34	78.71, 93.99
95% CI for failure rate		3.89, 31.66	2.19, 27.35	2.66, 32.36	6.01, 21.29
Cellulitis	*n*	11	11	13	35
48 to 72 h after randomization					
Success	*n* (%)	9 (81.8)	10 (90.9)	12 (92.3)	31 (88.6)
Failures	*n* (%)	2 (18.2)	1 (9.1)	1 (7.7)	4 (11.4)
thereof indeterminates	*n*	1	1	1	3
95% CI for success rate		48.22, 97.72	58.72, 99.77	63.97, 99.81	73.26, 96.80
95% CI for failure rate		2.28, 51.78	0.23, 41.28	0.19, 36.03	3.20, 26.74
EOT					
Success	*n* (%)	10 (90.9)	10 (90.9)	12 (92.3)	32 (91.4)
Failures	*n* (%)	1 (9.1)	1 (9.1)	1 (7.7)	3 (8.6)
thereof indeterminates	*n*	1	1	1	3
95% CI for success rate		58.72, 99.77	58.72, 99.77	63.97, 99.81	76.94, 98.20
95% CI for failure rate		0.23, 41.28	0.23, 41.28	0.19, 36.03	1.80, 23.06
STFU					
Success	*n* (%)	9 (81.8)	8 (72.7)	11 (84.6)	28 (80.0)
Failures	*n* (%)	2 (18.2)	3 (27.3)	2 (15.4)	7 (20.0)
thereof indeterminates	*n*	1	2	2	5
95% CI for success rate		48.22, 97.72	39.03, 93.98	54.55, 98.08	63.06, 91.56
95% CI for failure rate		2.28, 51.78	6.02, 60.97	1.92, 45.45	8.44, 36.94

a Exact binomial 95% confidence intervals (CI) are presented. Indeterminates are considered failures. Percentages are based on the number of patients with assessments available in the respective stratum at the respective visit.

### Safety.

Overall, of the 324 patients in the safety population (Fig. 1), 144 experienced at least one treatment-emergent adverse event (TEAE) (Table 9). More patients in the HD afabicin and vancomycin/linezolid groups experienced a TEAE than in the LD afabicin group (45.8%, 46.7%, and 40.9%, respectively). The most frequently reported TEAE in each treatment group was headache, which was experienced by a higher percentage of patients in the HD afabicin group (17.8%) than in the LD afabicin group (9.1%) and vancomycin/linezolid group (10.3%). The percentages of patients that experienced a treatment-emergent infusion site reaction were similar between treatment groups (4.5% [5/110], 4.7% [5/107], and 4.7% [5/107] in the LD afabicin, HD afabicin, and vancomycin/linezolid groups, respectively). Of the patients with TEAEs, most (97.9%, 141/144) had mild or moderate events. Three patients (all in the afabicin treatment groups) had severe TEAEs: two were considered not related to study medication (one case of cellulitis and one case of heroin overdose [considered a serious adverse event [SAE]), and one patient in the LD afabicin group experienced nephrolithiasis and renal colic, both of which were related to study medication.

**TABLE 9:** Overview of adverse events (safety population)^*a*^

Variable	Value for treatment group		
	LD afabicin (80/120 mg BID) (*n* = 110)	HD afabicin (160/240 mg BID) (*n* = 107)	Vancomycin/linezolid (BID) (*n* = 107)
Patients with ≥1 TEAE, *n* (%)	45 (40.9)	49 (45.8)	50 (46.7)
Severe	1 (0.9)	2 (1.9)	0
Moderate	12 (10.9)	13 (12.1)	19 (17.8)
Mild	32 (29.1)	34 (31.8)	31 (29.0)
Patients with TEAE related to i.v. administration, *n* (%)	10 (9.1)	21 (19.6)	15 (14.0)
Severe	1 (0.9)	0	0
Moderate	2 (1.8)	5 (4.7)	1 (0.9)
Mild	7 (6.4)	16 (15.0)	14 (13.1)
Patients with TEAE related to oral administration, *n* (%)	16 (14.5)	21 (19.6)	19 (17.8)
Severe	1 (0.9)	0	0
Moderate	2 (1.8)	5 (4.7)	5 (4.7)
Mild	13 (11.8)	16 (15.0)	14 (13.1)
Patients with most commonly reported qaTEAEs (≥5% in any group), *n* (%)			
Diarrhea	3 (2.7)	3 (2.8)	6 (5.6)
Nausea	7 (6.4)	9 (8.4)	7 (6.5)
Vomiting	1 (0.9)	6 (5.6)	2 (1.9)
Abscess	3 (2.7)	6 (5.6)	0
Headache	10 (9.1)	18 (16.8)	9 (8.4)
Patients with SAEs, *n* (%)	2 (1.8)	1 (0.9)	1 (0.9)
Deaths, *n* (%)	0	1 (0.9)	0

a AE, adverse event; BID, twice daily; i.v., intravenous; HD, high dose; LD, low dose; *n*, total number of patients; SAE, serious adverse event; TEAE, treatment-emergent adverse event.

Cardiac TEAEs were reported by two patients in the vancomycin/linezolid group (angina pectoris and nodal arrhythmia) and one patient each in the LD afabicin group (sinus tachycardia, considered unrelated to study medication) and the HD afabicin group (mild QT interval prolongation considered related to study medication). A postdose maximum QT interval with Fridericia’s correction (QTcF) of >500 ms was detected in two patients in the vancomycin/linezolid group (none in the afabicin groups) and of 480 to ≤ 500 ms in three patients in each of the HD afabicin and vancomycin/linezolid groups (none in the LD afabicin group).

No hepatic TEAEs were reported; however, seven patients experienced elevated liver enzymes, mostly at >2 times the upper limit of normal (2× ULN). None of the patients in the HD afabicin group had an alanine transaminase (ALT) concentration at >3× ULN; however, ALT at >3× ULN was detected in three patients in the LD afabicin group and one patient in the vancomycin/linezolid group. One patient in each treatment group had an aspartate transaminase (AST) concentration at >3× ULN. None of the patients in the study had a total bilirubin count of >1.5× ULN, and there were no cases that met Hy’s law.

Four patients experienced an SAE (Table 9) and discontinued study medication. Two patients in the LD afabicin group experienced moderately severe treatment-emergent SAEs of worsening of the primary ABSSSI, one of which was considered related to the i.v. study medication (neither patient received oral study medication). Both patients had MRSA isolated from their lesions at baseline. One patient in the HD afabicin group experienced moderate bacteremia (baseline blood culture positive for S. aureus) which was considered not related to the study medication (blood sample taken prior to first dose of afabicin) or study procedure. One patient in the vancomycin/linezolid group developed moderate cellulitis during follow-up which was not related to the study medication (i.v. or oral) or study procedure.

One patient, with a history of drug abuse and hepatitis C and who was on diamorphine at the time of randomization, died on day 3 of the study due to a heroin overdose. The patient was in the HD afabicin group and had received two doses of i.v. afabicin and one dose of oral afabicin. The investigator did not consider this to be related to the study medication or study procedure, and no autopsy was performed.

## DISCUSSION

In this phase 2 trial, afabicin, administered BID at two dose levels, was noninferior to vancomycin/linezolid in the treatment of patients with ABSSSI due to staphylococci. Furthermore, both dosing levels of afabicin were well tolerated.

The primary efficacy endpoint used in this study of lesion response at 48 to 72 h is part of the FDA guidelines for the development of drugs for the treatment of ABSSSI (16). While the highest early clinical response (ECR) rate observed in the mITT population was for the LD afabicin group (94.6%), rates for HD afabicin and vancomycin/linezolid were 90.1% and 91.1%, respectively. Overall, these ECR rates compared favorably with those of ceftaroline, delafloxacin, iclaprim, linezolid, and tedizolid (17–22). Rates of investigator-assessed clinical success at EOT and STFU were also high (>87% and >83%, respectively). At the STFU time point, clinical success was lower in the LD and HD afabicin groups (84.8% and 83.5%, respectively) than in the vancomycin/linezolid group (92.1%). However, of the patients who missed the STFU visit in the mITT population, seven in the afabicin group and one in the vancomycin/linezolid group had an outcome of clinical success at EOT. Furthermore, clinical success rates were comparable between treatment groups among patients who reached STFU without major protocol deviations, that is, in the PP population (97.0%, 98.2%, and 100% for LD afabicin, HD afabicin, and vancomycin/linezolid groups, respectively). All patients in the present study with polymicrobial infections involving a nonstaphylococcal pathogen in the afabicin groups showed an early clinical response, suggesting that in the context of polymicrobial infections with bacteria that are not susceptible to the agent, specifically targeting the staphylococcal pathogen with afabicin led to a positive outcome of the infection.

The efficacy of afabicin desphosphono, the active moiety of afabicin, has been previously demonstrated by Hafkin et al. (15) in a phase 2 study of oral afabicin desphosphono for the treatment of patients with ABSSSI. Following oral administration of afabicin desphosphono at 200 mg BID, 82.9% of patients in the microbiologically evaluable population achieved ≥20% decrease in the area of erythema on day 3 (15), which was lower than the early clinical response rate reported in the current study. The differences between the two studies include the initial routes of administration and different formulations (afabicin versus afabicin as the desphosphono salt) and the use of two dosing regimens, with no requirement for fasting, in the current study. Importantly, these factors are not expected to impact the pharmacokinetic (PK) profile between the studies, as phase 1 PK studies have demonstrated that afabicin (i.v. infusion or oral dosing) is rapidly converted to the active moiety, afabicin desphosphono (time to maximum concentration of drug in serum [*T*_max_] at 2 to 4 h postdose) (14). Of note, the 240 mg BID afabicin (HD afabicin) dose regimen represents approximately 200 mg BID afabicin desphosphono, which was the dose tested in the previous ABSSSI study (15). Taken together, the two phase 2 studies have shown that the active moiety of afabicin is efficacious in the treatment of patients with ABSSSI.

The development of afabicin has led to i.v. and oral formulations of the antibiotic. Following the minimum of two doses of i.v. afabicin, approximately two-thirds of patients were assessed by the investigator as ready to step down to oral therapy. The advantages of an early change to oral therapy include the opportunity for an earlier hospital discharge, which benefits the patient as well as reducing health care costs (23). Furthermore, afabicin provides an opportunity for patients to step down from an i.v. to oral formulation of the same antibiotic, which is thought to be less complicated than other strategies (24). This is not an option for a number of agents approved for the treatment of ABSSSI, such as daptomycin, ceftaroline, dalbavancin, and oritavancin, for which oral formulations are not available, and vancomycin, which is not systemically useful (1). Afabicin is therefore a promising agent for the treatment of serious ABSSSI due to staphylococci that require i.v. therapy.

Risk factors for treatment failure among patients with an ABSSSI include drug/alcohol abuse, obesity, age, and involvement of difficult-to-treat pathogens (23). The demographic characteristics of patients in this study were generally well balanced across treatment groups; however, the study population included patients who were potentially difficult to treat due to their medical histories. For example, the average lesion size at baseline in this study exceeded 300 cm^2^; other clinical studies of ABSSSI patients have reported similarly large lesion sizes (17–19, 22, 25, 26). A large proportion of patients (≥82.2% in each treatment group) in this study had a history of drug abuse and therefore were more likely to have more advanced infections due to delays in seeking medical care (27). Furthermore, a high proportion of patients in the study were obese (body mass index [BMI] ≥ 30 kg/m^2^). Both drug abuse and obesity have been associated with recurrent emergency department visits (28). Despite the potential complications of a population with such comorbidities, 84.8% of randomized patients completed the study up to STFU. This figure compares favorably with other studies; for example, even with lower percentages of drug abusers (56.7% and 50.7%), the REVIVE 1 and 2 studies of i.v. iclaprim versus vancomycin for the treatment of ABSSSI due to Gram-positive pathogens reported comparable completion rates at the same time point (92.0% and 90.7%) (18, 19).

The IDSA guidelines for the treatment of severe ABSSSI include the use of vancomycin, linezolid, daptomycin, and telavancin (3); however, there are safety concerns related to these agents (4–7), underscoring the need for extending the available treatment options. In addition, more recently approved agents such as dalbavancin can be considered in this indication (29). As with all new agents, and especially those of a new chemical class, the safety of afabicin requires close scrutiny until more clinical data are available. However, in this study, afabicin, at both dosing levels, was generally well tolerated in patients with ABSSSI. The most commonly reported TEAE was headache. Treatment-emergent headaches were experienced by a higher proportion of patients in the HD afabicin group than in the LD afabicin and vancomycin/linezolid groups; all were mild or moderate in intensity. Comparison of the incidences of TEAEs between the LD and HD afabicin groups indicates that the safety profile of LD afabicin is marginally more favorable than that of HD afabicin. Four patients had SAEs, only one of which was considered related to i.v. (LD afabicin) study medication (exacerbation of skin infection; moderate in intensity). There were no deaths considered related to the study medication.

Afabicin is a potent inhibitor of staphylococcal FabI. In contrast to the broad-spectrum antibiotics, this selective spectrum of activity is expected to reduce the impact of afabicin on the intestinal microbiota (30). Indeed, in the current study, treatment-emergent diarrhea was experienced by half the number of patients in both the LD and HD afabicin groups as in the vancomycin/linezolid group. Furthermore, data from a phase 1 drug-drug interaction study during which 16 healthy subjects received oral afabicin (240 mg BID for 20 days) showed no impact of the agent on gut microbiota richness and diversity (31). Taken together, these studies indicate that owing to its narrow-spectrum activity, afabicin has the potential to eradicate pathogens while preserving commensal microbiota. The use of afabicin therefore has the potential to reduce the incidence of complications caused by microbiota dysbiosis, such as antibiotic-induced colitis or candidiasis, which can occur following broad-spectrum antibiotic therapy (11, 12, 32).

In conclusion, this study has shown that afabicin is efficacious and well tolerated in the treatment of ABSSSI due to staphylococci. *In vitro* studies have demonstrated that environments rich in fatty acids can favor the emergence of S. aureus variants that are resistant to afabicin desphosphono (33, 34). However, the results of the present study indicate that targeting FabI appears to be a valid approach in the ABSSSI setting. The availability of both i.v. and oral formulations of afabicin offers the possibility of using the same agent when changing from i.v. to oral treatment, which is advantageous when the patient is responding to treatment. The narrow spectrum of activity of afabicin is not only beneficial for the patient but also well aligned with antimicrobial stewardship, as it is believed that preservation of gut microbiota may also reduce spread of antibiotic resistance (35). Both the efficacy and safety data from this study support further development of afabicin for the treatment of ABSSSI and potentially pave the road for treatment of other types of staphylococcal infections such as bone and joint infections (36).

## MATERIALS AND METHODS

### Study design.

This was a multicenter, randomized, parallel-group, double-blind, and double-dummy phase 2 study to evaluate the efficacy, safety, and tolerability of i.v. and oral afabicin compared with i.v. vancomycin and oral linezolid in the treatment of clinically documented ABSSSI due to staphylococci susceptible or resistant to methicillin (ClinicalTrials registration number NCT02426918; https://clinicaltrials.gov/ct2/show/NCT02426918).

### Main inclusion criteria.

Patients eligible for inclusion were between 18 and 70 years of age and of either sex. Patients had a clinically documented ABSSSI (specifically, wound infection, cutaneous abscess, burn, or cellulitis) that was suspected or documented to be caused by a staphylococcal pathogen by either Gram staining showing Gram-positive cocci in clusters or a registered rapid diagnostic test. Patients had ABSSSI that were accompanied by clinical signs of erythema, edema, or induration measuring at least 75 cm^2^. The primary infected lesion had to show at least two of the following: significant pain or tenderness to palpation, purulent or seropurulent drainage or discharge, fluctuance, and/or heat or localized warmth. Patients had at least one of the following signs and symptoms of systemic inflammation or complicating factors: documented or reported fever of ≥38.0°C, white blood cell (WBC) count of >10,000 cells/mm³, >15% immature neutrophils irrespective of total WBC, local or regional lymphadenopathy, or elevated C-reactive protein. Patients who had received an antibiotic with activity against Gram-positive cocci within the 14 days preceding randomization were included if they met one of the following criteria: their causative Gram-positive pathogen from the ABSSSI lesion was *Staphylococcus* that was resistant *in vitro* to the antibiotic(s) administered (with clinical progression), they had documented failure to previous ABSSSI antibiotic therapy, or they had a single dose or a single course of short-acting antibiotic (4- to 6-h half-life) with potent antistaphylococcal activity within 24 h of randomization (limited to 25% of patients randomized).

### Main exclusion criteria.

Prior exposure to afabicin or afabicin desphosphono precluded enrollment into the study, as did any Gram-positive antibacterial therapy during the preceding 14 days or any other investigational medication during the preceding month (with some exceptions, as defined in the inclusion criteria). Patients excluded from the study were as follows: those with advanced disease with infected nonhealing wounds in peripheral sites, an abscess not drained or a wound infection involving foreign material not removed within 24 h of starting study medication, infected abdominal wounds that were unable to be surgically closed, necrotizing or gangrenous infections, or infected bites, or those with the primary site of infection on a limb that was likely to need amputation during the study. Patients with a primary infection (including erysipelas) due to suspected or documented streptococci or infection with a Gram-negative pathogen without concomitant staphylococcal infection or with a pathogen that was nonsusceptible to either study medication were also excluded, as were patients with sepsis or a nonskin source of infection and those not expected to survive for at least 60 days.

### Procedures.

Patients were randomized in a 1:1:1 ratio to receive either afabicin i.v. 80 mg BID followed by oral afabicin 120 mg BID (low dose [LD] afabicin), afabicin i.v. 160 mg BID followed by oral afabicin 240 mg BID (high dose [HD] afabicin), or vancomycin i.v. 1 g or 15 mg/kg BID followed by oral linezolid 600 mg BID (vancomycin/linezolid). Patients received their first dose of study medication on day 1 of the study. Following two doses of i.v. treatment, they were assessed by the investigator and were switched to oral treatment on day 2 if the acute toxicity of infection had resolved (resolution of fever, reduced/stable lesion size), the patient could tolerate fluids and a regular diet, and the investigator confirmed the patient no longer needed i.v. treatment. If needed, patients continued with i.v. dosing until they were ready for the switch to oral dosing. Treatment with study medication (i.v. and oral dosing) lasted between 7 days (14 doses; minimum treatment period for the patient to be evaluable) and 10 days (20 doses; maximum treatment period). Patients were assessed at baseline, i.e., at 48 to 72 h after randomization, within 24 h of the last dose (end of treatment [EOT]), and 7 to 14 days after EOT (short-term follow-up [STFU]).

During the screening period (within 48 h prior to randomization), the following were obtained for each patient: informed consent, eligibility verification, medical history, and demographic data. Two blood cultures were obtained from each patient at screening, and blood cultures were repeated if the patient remained febrile for >48 h. If they were positive for a pathogen, further blood cultures were obtained at least every 48 h until negative. If the patient’s repeated 48-h sample was positive for the baseline pathogen, he/she was to be discontinued from the study and would be considered a failure for primary endpoint. The patient would then be offered an alternative antibiotic treatment. If the patient’s blood cultures at any point after 48 h became positive for the baseline pathogen, he/she would be discontinued from the study and considered a failure. Lesions were assessed for area of erythema, edema/swelling, and induration at screening, 48 to 72 h postrandomization, at EOT, and at STFU. Lesion samples (including purulent wound exudates, skin lesion biopsy specimens, tissue samples, and aspirates of abscess cavities) were collected at screening for microbiological culture, Gram staining, identification, and susceptibility testing. After screening, lesion samples for microbiological assessment were taken only from wounds that had not healed and were not taken after day 3 unless there was a relapse.

All blood and lesion samples collected for microbiology were processed and analyzed by local laboratories according to their routine procedures. These analyses included Gram staining, species isolation and identification, and susceptibility testing. All clinically relevant bacterial isolates were shipped to the central laboratory for confirmation of species identification by matrix-assisted laser desorption ionization–time of flight mass spectrometry (MALDI-TOF MS) and susceptibility testing according to CLSI guidelines (37, 38) and for molecular characterization of resistance and virulence genes by PCR and pulsed-field gel electrophoresis (PFGE) typing when appropriate. Data from the central laboratory were used in this study.

Concomitant medications were recorded daily until EOT and at STFU. The protocol was amended such that amoxicillin was administered to all patients with cellulitis, irrespective of treatment group. Nonstudy antibiotics with little or no activity against *Staphylococcus* spp. were permitted throughout the study, as was a single dose or a single course of short-acting antibiotic (4- to 6-h half-life) with potent antistaphylococcal activity within 24 h of randomization (limited to 25% of patients randomized). Treatment with vancomycin was allowed 6 to 12 h prior to screening (no later than 7.5 days prior to screening in patients with renal dysfunction).

### Analysis populations.

Four analysis populations were defined. The intent-to-treat (ITT) population included all randomized patients. The microbiological intent-to-treat (mITT) population comprised all randomized patients who had an identified baseline staphylococcal pathogen (S. aureus and/or a pathogenic coagulase-negative *Staphylococcus* [CoNS], including S. epidermidis, S. haemolyticus, and S. lugdunensis]) and received at least one dose of study drug. The per-protocol (PP) population comprised all patients in the mITT population who completed the study up to STFU without any major protocol deviations. The safety population comprised all patients who received at least one dose of study drug.

### Efficacy outcomes.

The primary efficacy endpoint was an early clinical response rate at 48 to 72 h following randomization in the mITT population, as specified in the FDA guidelines (16). Responders were patients whose primary ABSSSI lesion involving erythema, edema, or induration had decreased by ≥20% in area from baseline. Nonresponders were patients in the following categories: did not meet the criteria for clinical responders, required systemic concomitant antibiotic therapy that was potentially effective against the baseline staphylococcal pathogen, required unplanned incision and drainage of the ABSSSI within 48 to 72 h following randomization, required unplanned major surgery due to failure of study medication, and death prior to evaluation of the primary efficacy endpoint.

Secondary efficacy endpoints were clinical and microbiological outcomes. Clinical outcome (success or failure) was based on the investigator’s assessment of the patient’s signs and symptoms of infection in the mITT and PP populations at 48 to 72 h following randomization, EOT, and STFU. Clinical success was defined as the resolution or near resolution of most disease-specific signs and symptoms, no new sign, symptoms, or complications, and no requirement for further antibiotic therapy for the treatment of the original site of infection at EOT or STFU. Clinical failures were the following: patients who did not meet all the criteria for clinical success, patients in whom unplanned incision and drainage of the ABSSSI was performed within 48 to 72 h following randomization or in whom unplanned major surgery was required due to failure of study medication, and patients who developed osteomyelitis after baseline.

Microbiological outcomes were determined at 48 to 72 h following randomization, EOT, and STFU for all patients in the mITT population. Documented eradication was defined as the absence of baseline pathogens in follow-up cultures of the original site of infection. Conversely, documented persistence was the presence of baseline pathogens in follow-up cultures of the original site of infection. Presumed eradication and presumed persistence were assigned in cases where samples were not available for culture (lesion samples were not taken from wounds that had healed) and involved an investigator assessment of clinical outcome. A superinfection, at 48 to 72 h postrandomization and EOT, was defined as a new pathogen at the original site of infection during treatment in the presence of signs and/or symptoms of infection. A new infection at STFU was defined as a new pathogen at the original site of infection after treatment, in the presence of signs and/or symptoms of infection.

### Safety.

Treatment-emergent adverse events (TEAEs) and serious adverse events (SAEs) were reported by treatment group and were evaluated for severity and relationship to study medication (by i.v. treatment, oral treatment, and study procedure separately). All adverse events (AEs) were monitored until they were resolved, any abnormal laboratory values had returned to baseline levels or stabilized, or until there was a satisfactory explanation for the changes observed. An SAE was defined as any untoward medical occurrence that, at any dose, resulted in death, was life-threatening, required inpatient hospitalization, or resulted in persistent or significant disability/incapacity.

Study procedures for safety assessments included the following: recording of AEs (every visit), clinical laboratory tests (hematology, serum chemistries, and coagulation tests within 24 h of screening, 48 to 72 h postrandomization, and at EOT), vital signs (blood pressure, pulse measurements, body temperature, and respiration rate at screening, before the first and second i.v. doses, 48 to 72 h postrandomization, after the first oral dose and last morning dose, at EOT/early termination [ET], and STFU), electrocardiograms (at screening, 48 to 72 h postrandomization and prior to and 2 to 4 h after the last morning dose at EOT), and physical examination findings (at screening, EOT, and STFU).

### Statistics.

The study was designed to demonstrate noninferiority between afabicin and vancomycin/linezolid at the primary efficacy endpoint. A sample size of at least 231 patients in the mITT population was required to demonstrate noninferiority using a noninferiority margin of 15%, a 2-sided type I error of 5% and power of 80%, and when the early clinical response rate was assumed to be 87.5% in all treatment groups. Noninferiority was established if the upper bound of the two-sided 95% confidence interval for the difference in ECR rates (afabicin ECR rate minus vancomycin/linezolid ECR rate) was <0.15.

For secondary outcomes and safety assessments, descriptive analyses were performed in the mITT, PP, and safety populations, respectively.

### Ethical conduct.

The protocol and informed consent form were reviewed and approved by an institutional review board or independent ethics committee at each study center prior to study initiation, and the study was conducted according to the protocol and any subsequent amendments. Informed consent was obtained from each patient before any study-related investigations were performed. The study was conducted according to the ethical principles of good clinical practices as defined in the U.S. Code of Federal Regulations, the International Council for Harmonisation of Technical Requirements for Pharmaceuticals for Human Use (ICH) E6 Good Clinical Practice, and local ethical and legal requirements.
